# A new Surgical Technique: Transvesical Prostate Resection

**DOI:** 10.1590/S1677-5538.IBJU.2018.0488

**Published:** 2019-12-17

**Authors:** Hakan Türk, Erkan Arslan

**Affiliations:** 1 Department of Urology, Evliya Celebi Training and Research Hospital, Kutahya, Turkey;; 2 Department of Urology, Harran University Medical School, Sanliurfa, Turkey

## Abstract

**Objectives:**

Surgical treatment is indicated in patients where medical therapy fails to prove beneficial or in patients who develop complications related with bladder outlet obstruction. In our study, we developed a new surgical technique which can be defined as Transvesical Resection of Prostate (TVRP) without using the urethra. This method was previously described in our articles (1).

**Materials and Methods:**

A 62-years-old male patient, using an alpha blocker agent for 5 years, reported increased discomfort with urination. His findings were as follows: PSA: 1.2 ng/dL, prostate volume: 45 cc, digital rectal examination: benign, IPSS: 30, QoL: 5, Qmax: 6, urine volume: 225 cc, post-mictional residue: 65 cc. Eventually the patient was informed and prostate resection decision was made.

**Results:**

Suprapubic catheter was removed 1 day after surgery and the patient was discharged. Urethral catheter was removed 4 days after urine output became clear. No complications developed after the operation. At postoperative 1st month, Qmax was 22, urine volume was 260 cc, post-mictional residue was 40 cc, IPSS was 8, QoL was 1, and the pathology was benign prostate tissue.

**Conclusions:**

Urethral stricture is one of the most important postoperative complications of TURP. The incidence of urethral stricture is reported between 2.2% and 9.8% in different series (2–5). In this technique which we developed, urethra is not used and prostate is removed through the bladder, similar to open prostatectomies. For this reason, we suggest that it has an advantage over TURP, regarding urethral stricture development.

## ARTICLE INFO

**Available at: http://www.intbrazjurol.com.br/video-section/20180488_Turk_et_al**

**Int Braz J Urol. 2019; 45 (Video #X): 1279-80**
